# Efficacy comparison of trifocal bone transport using unilateral external fixator for femoral and tibial bone defects caused by infection

**DOI:** 10.1186/s12893-022-01586-z

**Published:** 2022-04-12

**Authors:** Kai Liu, Yanshi Liu, Feiyu Cai, Chenchen Fan, Peng Ren, Aihemaitijiang Yusufu

**Affiliations:** Department of Trauma and Microreconstructive Surgery, The First Affiliated Hospital of Xinjiang Medical University, Ürümqi, 830054 Xinjiang China

**Keywords:** Bone defect, Bone transport, External fixator, Ilizarov method, Infection

## Abstract

**Background:**

This study aimed to evaluate the clinical and functional outcomes of patients with femoral and tibial critical-sized bone defect (CSBD) treated by trifocal bone transport using the Ilizarov method.

**Methods:**

From March 2011 and January 2017, clinical and radiographic data of patients with CSBD (> 6 cm) caused by infection were documented and analyzed. Patients were divided into the femur group (n = 18) and tibia groups (n = 21) according to the location of bone transport. The bone and functional outcomes were evaluated according to the Association for the Study and Application of the Method of the Ilizarov (ASAMI) criterion, and postoperative complications were evaluated by Paley classification.

**Results:**

A total of 39 patients were managed by the trifocal bone transport for the femur (n = 18) or tibia (n = 21) bone defects with a mean follow-up time of 26.1 months (range 17–34 months). Eighteen femurs and 21 tibias with a mean distraction regenerate length (DRL) of 8.3 cm (range 6–13 cm) and 7.5 cm (range 6–11 cm) respectively. Infection was eradicated in all patients, and the total bone union was received in all cases (100%). Statistical difference of bone grade (excellent/good/fair/poor, 3/11/3/1 vs 2/13/4/2, P < 0.05), and function grade (excellent/good/fair/poor, 3/14/1/0 vs 4/13/3/1, P < 0.05) were respectively observed between the femur group and tibia group. The excellent and good rate of bone (femur vs tibia, 77.8% vs 71.4%), and function grade (femur vs tibia, 94.4% vs 80.9%) was higher in the femur group than the tibia. The rate of complication in the femur group was lower than in the tibia (femur vs tibia, 94.4% vs 76.2%). One femur and five tibias were performed additional surgery for delayed union and axial deviation.

**Conclusions:**

The trifocal bone transport using the unilateral external fixator was a practical method in the management of CSBD in the lower extremity. The BUT and EFI of the femur group were shorter than the tibia. Although the complications noted were more frequent on the femur, these were mostly minor.

## Background

The critical-sized bone defect (CSBD, > 6 cm) caused by trauma, osteomyelitis, and tumor resection, still leaves a tricky problem for orthopedic clinicians [1–4]. The CSBD usually combines with complex comorbidities, such as draining sinus, poor soft tissue coverage, joint stiffness, limb deformity, or drug-resistant polymicrobial infection. Therefore, multiple-stage surgery is generally required for the management of the above problems, which may be an invasive, time-consuming, and high financial burden. Once the previous treatment failed, the recurrence of infection may take place, which brings out the new injurious changes for the affected limbs.

The generally accepted principle in the treatment of CSBD is radical debridement, and bone regeneration using stable fixation, which equalizes the length and alignment force of the limb to obtain the satisfactory recovery of function. With this conception, several methods for the reconstruction of bone defects in the extremities have been applied for patients with critical bone defects, including the Ilizarov technique, vascularized fibular transplantation, autologous cancellous bone grafting, and Masquelet technique [5–8]. The advantages of trifocal bone transport [9], have been proposed by previous studies to reduce postoperative complications, such as long external fixation time, pin tract infection, and hypoplastic bone regeneration [10–14]. However, few studies evaluate its differences and clinical efficacy in the treatment of femoral and tibial CSBD.

Hence, the purpose of this study was to evaluate the clinical outcomes and differences in complications of the trifocal bone transport in the management of infectious femoral and tibia defects.

## Materials and methods

### Study design

After receiving the written informed consent from participants and approval from our hospital’s Ethics Committee, the medical records and radiographs were evaluated retrospectively of all patients from March 2011 and January 2017. Inclusion criteria are as follows [15, 16]: femoral or tibial CSBD (defined as bone defect > 6 cm [10] caused by infection; sinus tract or abscess of affected limbs; positive intraoperative culture or histology supporting a deep infection; CSBD treated by trifocal bone transport. Patients were excluded for those younger than 18 years old, incomplete medical records, poor compliance, or follow-up time less than 20 months [16]. All patients were performed by the suitable mode (adjusted to the location of the bone defects) of trifocal bone transport using a unilateral external fixator (Orthofix limb reconstruction system, Verona, Italy) after radical debridement.

The demographic data, previous surgical and medical treatment, comorbidities, antimicrobial utilization, biopsy or culture results of secretions, and intraoperative data were documented. The index of inflammatory was recorded, such as C-reactive protein (CRP), white blood cell (WBC), procalcitonin, and erythrocyte sedimentation rate (ESR). The degree of bone infection was evaluated by Cierny and Mader’s (CM) classification. The sensitive antibiotics were given to all patients intravenously for 2 weeks depending on the bacteria isolated.

### Surgical procedure

Patients were positioned supine on the radiolucent table, and spinal anesthesia was performed. According to our previous study [15, 16], the affected limb's necrotic bone and soft tissue were removed firstly until the residual bone showed evidence of punctate cortical hemorrhage (paprika sign). Intraoperative specimens of the infected area were collected and sent for bacterial culture and susceptibility testing to guide the surgeon in the selection of appropriate postoperative antibiotics. The surgical area was flushed with 0.9% saline under low pressure. The gloves of all participating surgeons and surgical instruments were then replaced. Antibiotic-impregnated cement spacer (5 g vancomycin per 40 g gentamicin-loaded bone cement, Heraeus, Hanau, Germany) equal in length to the bone defect was filled into the defect to receive a high level of local antibiotic concentrations. Hereafter, the external fixators were placed on the proximal and distal femur or tibia in an anterolateral position parallel to the respective joint. Three 4.5-mm-diameter Schanz screws were inserted at the fragment of the proximal and distal femur or tibia, and two same Schanz screws were respectively inserted at the transport bone fragment under the guidance of the intraoperative radiography machine [16]. Simultaneously, the desired length and alignment of the femur or tibia were maintained. These screws were directed at right angles to the anatomical axis of the femur. After the external fixator sliding clamps were assembled and the external frame debugged to parallel to the axis of the femur, the minimally invasive osteotomy was performed by Gigli saw. Depending on the size of the soft tissue loss, tension-free direct suture, keystone flap, or free vascularized flap were performed to repair it. Removal of the spacer was conducted as the infection was under control, which was determined by laboratory parameters such as WBC, CRP, and ESR.

### Postoperative management

Distraction osteogenesis was started after a latent period of 7 days. The proximal fragment and the distal fragment were distracted four times per day at a rate of 0.25 mm respectively until the two fragments converged. On the 2nd postoperative day, postoperative rehabilitation was encouraged to start by performing active and passive knee range of motion (ROM) exercises without weight-bearing. Weight-bearing walking was encouraged for patients at the whole consolidation stage. The pin tract care was instructed to the patients to prevent the pin tract infection [15], such as washing the pin tract daily using a swab with 0.9% saline. Subsequently, radiography, WBC, ESR, and CRP were examined at 1, 3, 6, 9, 12, 18, and 24 months after bone transport [16]. The pain of affected limbs and the psychological status of the patients were monitored closely.

### Data collection and outcome evaluation

The Association for the Study and Application of the Method of Ilizarov (ASAMI) criteria was applied to assess the bone and functional results, and complications evaluated by Paley classification (Minor was defined as not required additional surgery, and major was defined as either resolved with additional surgery or remained unresolved) [10, 17]. The incidence and differences of complications in both femoral and tibial bone transport were recorded and compared.

### Statistical analysis

Data were input in a Microsoft Excel spreadsheet (Redmond, WA, USA), presented as frequencies and percentages, and then analyzed by the SPSS 20.0 software package (Chicago, IL, USA). Continuous variables were analyzed by independent-samples T-tests and expressed as the mean and standard deviation. And categorical variables were analyzed by the chi-square test, expressing as the number. Statistical significance was P < 0.05.

## Results

A total of 39 patients who met the inclusion criteria were managed by the trifocal bone transport for the femur (n = 18) or tibia (n = 21) bone defects with a mean follow-up time of 26.1 months (range 17–34 months) in our hospital. There were 26 males and 13 females with a mean age of 46.7 years (range 20–52 years). Eighteen femurs and 21 tibias with a mean distraction regenerate length (DRL) of 8.3 cm (range 6–13 cm) and 7.5 cm (range 6–11 cm) respectively, were included in this study. Bone defects in all patients were the result of radical debridement procedures after trauma or osteomyelitis. According to the Cierny and Mader’s (CM) classification, there were 29 patients in type III, and 10 patients in type IV, which positive bacteria isolated was received in 33 cases (84.6%). In detail, twenty patients (57.8%) were infected with *S. aureus*, 7 (21.2%) in *P. cuprina*, and 6 (18.1%) with *E. coli*. Demographics and intraoperative data of the two groups were summarized in Table 1.

**Table 1:** Comparison of the main indicators of the two groups

	Femur (n = 18)	Tibia (n = 21)	*z* or *t*	P value
Age (years)	46.29 ± 6.29	47.05 ± 7.29	− 0.338	0.737
Gender (male, female)	13 M, 5 F	13 M, 8 F	− 0.357	0.721
DRL (cm)	8.37 ± 3.48	7.57 ± 2.73	1.768	0.086
Operating time (min)	159.24 ± 13.28	149.91 ± 7.22	2.587	0.016
Surgical bleeding volume (ml)	172.79 ± 15.81	139.44 ± 12.35	7.053	< 0.001
Duration of distraction stage (day)	40.41 ± 8.67	45.45 ± 5.75	− 2.115	0.042
CT (day)	231.54 ± 3.31	250.46 ± 2.99	18.110	< 0.001
BUT (day)	323.72 ± 5.66	344.25 ± 3.69	12.802	< 0.001
EFT (day)	334.49 ± 8.54	344.64 ± 3.64	− 4.555	< 0.001
EFI (days/cm)	55.96 ± 2.96	65.02 ± 1.29	11.677	< 0.001
Duration of disease (month)	22.10 ± 5.16	20.63 ± 2.44	1.078	0.293
Follow-up time (month)	27.75 ± 4.06	27.34 ± 3.38	0.083	0.086

*BUT* bone union time; *CT* consolidation time; *DRL* distraction regenerate length; *EFI* external fixation index; *EFT* external fixation time

Infection was eradicated in all patients, and the total bone union was received in 39 of 39 cases (100%). Soft tissue loss was covered with the direct sutures of appropriate tension in 31 patients (79.5%), local propulsive skin flaps in 6 patients (15.4%), and vascularized free flaps in 2 patients (5.1%). Skin or flaps necrosis was not observed. There were no significant differences in age, gender composition, DRL, duration of disease, and follow-up time between the two groups (P > 0.05). In contrast, there was a significant statistical difference (P < 0.05) between the femur group and the tibia group among the operating time, surgical bleeding volume, duration of distraction stage, consolidation time (CT), bone union time(BUT), external fixation time (EFT), external fixation index (EFI).

**Fig. 1:**
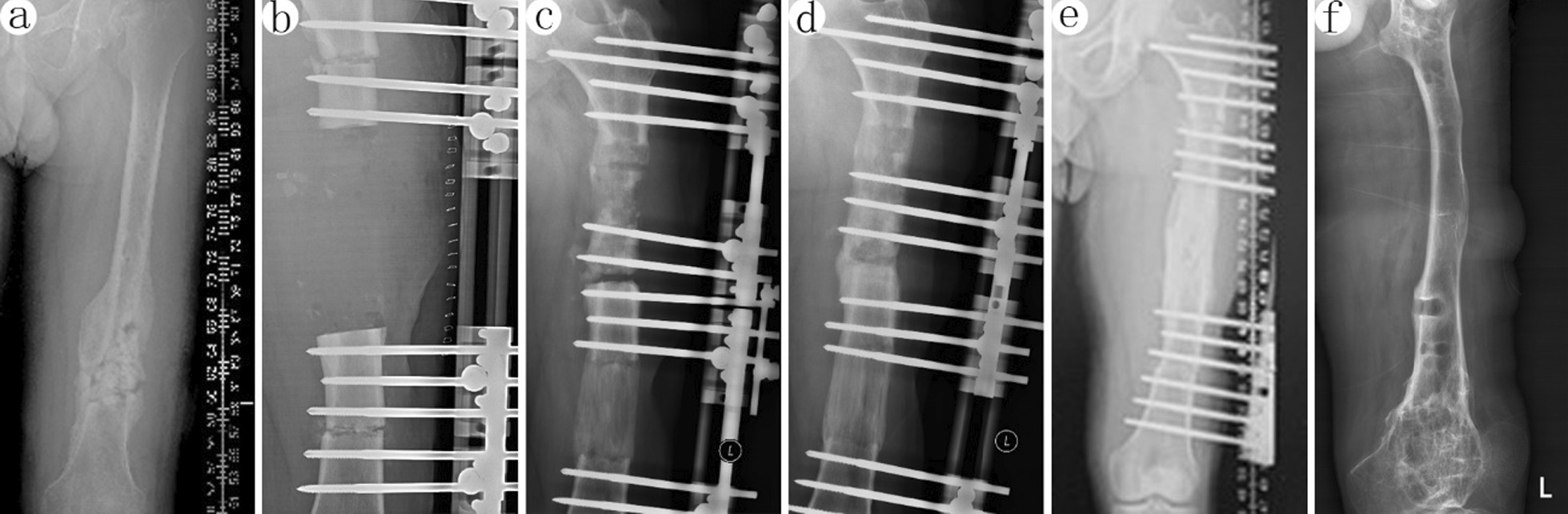
39-year-old male patient with posttraumatic osteomyelitis of the left femur was treated with trifocal bone transport from both side to docking site using Orthofix external fixator. **a** X-ray graph before treatment. Lesions on the sides of the middle and lower part of the femur. **b** After debridement, the bone defect reached almost 14 cm. The Orthofix external fixator was used to perform the distal and proximal biplane osteotomy. **c** The X-ray graphs at 40 days after surgery. The force line was available. **d** At 80 days, the butt joint healed. Regeneration zone growing well. **e** Bone healing of the osteotomy line was evident at 8 months. **f** X-ray film after removing the external frame at last follow-up

**Fig. 2:**
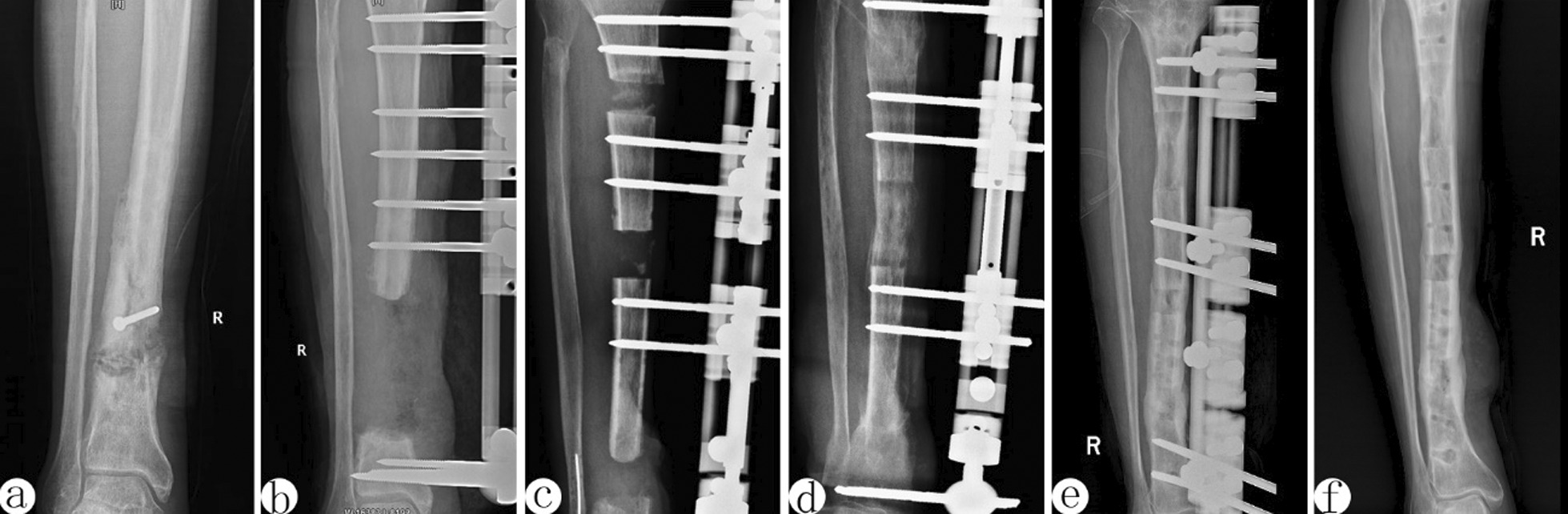
A 47-year-old female patient with posttraumatic osteomyelitis the right tibia and treated using Orthofix external fixator trifocal bone transport from proximal to distal. **a** X-ray graph before treatment. Lesions and internal fixator were on the sides of the middle and lower part of the tibia. **b** Segmental defect of the left tibia after debridement on X-ray graph. Excision of infection bone with 12 cm defect and application of Orthofix external fixation with trifocal bone transport. **c** The X-ray graphs at 40 days after surgery. The force line was available. **d** Bone healing of the osteotomy line was evident at 4 months. **e** Bone transport was completed with good regenerate consolidation and docking union was achieved and evaluated on the view of X-ray at 6 months. **f** Orthofix external fixator was removed with excellent bone union shown on the view of X-ray at 8 months after operation

Radiating foot pain occurred in 26 of 39 patients, which was relieved by slowing the distraction rate. Furthermore, pin tract infection occurred in 7 patients (4 femurs and 3 tibias), and three of them (Checketts and Otterburn classification IV) progressed to pin loosening, which was resolved by dressing change combined with oral antibiotics or the pin tract replacement surgery. Axial deviation (3 femurs and 2 tibias) was corrected by adjusting the sliding clamps of the external fixator radiologically under local anesthesia. Muscle contractures (2 femurs) were managed by tension-release surgery. Joint stiffness (2 femurs and 1 tibia), soft tissue incarceration (2 femurs and 1 tibia), and neurological injury (3 femurs and 4 tibias) were treated by rehabilitation training and physical electromagnetic wave therapy. Delayed unions (1 femur and 5 tibias) were successfully managed by autologous bone grafting at the docking site. The whole procedure of trifocal bone transport of femur and tibia described in this study was shown in Figs. 1, and 2.

The details of bone and functional outcomes at the last follow-up after removal of the external fixator were summarized in Table 2, which were evaluated according to the ASAMI criteria. Statistical difference of bone grade (excellent/good/fair/poor, 3/11/3/1 vs 2/13/4/2, P < 0.05), and function grade (excellent/good/fair/poor, 3/14/1/0 vs 4/13/3/1, P < 0.05) were respectively observed between the femur group and tibia group. The excellent and good rate of bone (femur vs tibia, 77.8% vs 71.4%), and function grade (femur vs tibia, 94.4% vs 80.9%) was higher in the femur group than the tibia. Additionally, the distribution of complications in the two groups was recorded in Table 3. The rate of complication in the femur group was lower than in the tibia (femur vs tibia, 94.4% vs 76.2%). According to Paley's classification, the distribution of minor (n = 26) and major (n = 7) complications were shown in Table 4. Minor complications of each patient in the femur and tibia group were 0.44 and 0.23 respectively (P < 0.001, Table 5), and major complications of each patient were 0.06 and 0.11 (P < 0.05). Complications such as sequelae and unresolved at the end of treatment were not observed.

**Table 2:** Outcomes of ASAMI scores in two groups

ASAMI	Location	Excellent	Good	Fair	Poor	Failure
Bone grade*	Femur	3	11	3	1	–
	Tibia	2	13	4	2	–
Function grade*	Femur	3	14	1	0	0
	Tibia	4	13	3	1	0

*Bone results* Excellent: Union, no infection, deformity < 7°, limb length discrepancy (LLD) < 2.5 cm; Good: Union plus any two of the following: the absence of infection, deformity < 7°, LLD < 2.5 cm; Fair: Union plus any one of the following: the absence of infection, deformity < 7°, LLD < 2.5 cm; Poor: Nonunion/refracture/union plus infection plus deformity > 7° plus LLD > 2.5 cm

*Functional results* Excellent: Active, no limp, minimum stiffness (loss of < 15°knee extension or < 15°ankle dorsiflexion) no reflex sympathetic dystrophy (RSD), insignificant pain; Good: Active, with one or two of the following: limb, stiffness, RSD, significant pain; Fair: Active, with three or all of the following: limb, stiffness, RSD, significant pain; Poor: Inactive (unemployment or inability to return to daily activities because of injury); Failure: Amputation

*P < 0.05

**Table 3:** The complications of two groups in the period of bone transport

Complication	Femur (n = 18)	Tibia (n = 21)
Pin tract infection	4	3
Muscle contractures	2	0
Joint stiffness	2	1
Axial deviation	3	2
Soft tissue incarceration	2	1
Neurological injury	3	4
Delayed union	1	5
Nonunion	0	0
Recurrence of infection	0	0

**Table 4:** The details of bone transport related complications

Complications	Minor	Major
Pin tract infection or pin loosening	6	1
Muscle contractures	2	0
Joint stiffness	3	0
Axial deviation	4	1
Soft tissue incarceration	2	1
Neurological injury	6	1
Delayed union	3	3
Nonunion	0	0
Recurrence of infection	0	0

**Table 5:** The treatment results of two group postoperative complications

	Femur	Tibia	P value
Docking site revision	1	5	–
Minor complications (per patient)	0.23	0.44	< 0.001
Major complications (per patient)	0.11	0.06	< 0.001
Sequelae (per patient)	–	–	–
Total surgeries (per patient)	4	7	–

## Discussion

This study aimed to evaluate the clinical outcomes and differences in complications of the trifocal bone transport in the management of femoral and tibia CSBD caused by infection. The rate of bone union was 100%. The BUT, CT, EFT, and EFI of the femur group were less than the tibia. The incidence of minor complications was significantly higher in the femur group, such as pin tract infection, and axial deviation. But the excellent and good rate of bone and function grade was higher in the femur group since the sufficient soft tissues and blood vessels to allow earlier postoperative rehabilitation and enhance the bone regeneration. Major complications were at higher risk of the tibia group, such as a delayed union. Internet questionnaire was a convenient and practical tool for orthopedic clinicians to timely and accurately monitor postoperative management.

Via previous studies [12, 14, 18–20], the Masquelet technique, vascularized fibular grafting, and autogenous or allogenic bone grafting have been reported about their efficacy in controlling infection and repairing the bone defects, but the disadvantages of these techniques are also not ignored, including complex microsurgery, risk of fracture, poor ability in correcting deformity, and complications of the donor site. Several improved methods have been used for reducing the incidence of complications caused by long EFT. Burghardt et al. [21] reported a matched case comparison of tibial lengthening over the nail (LON) with treatment using the traditional Ilizarov method, considering that LON reduced the EFT effectively. A meta-analysis published by Xu et al. [22] found that lengthening and then nailing (LATN) is superior to the conventional Ilizarov method in regards to the EFT and the CT. The advantages of the PRECICE (NuVasive Specialized Orthopedics, San Diego, CA, USA) magnetic intramedullary compression and distraction nail had created a new ideally option [23, 24], which combined the convenience of the intramedullary nail with the sustained compression for bone healing of distraction area. Moreover, nine patients with lower extremities’ bone defects treated by plate-assisted bone segment transport (PABST) were reported by Olesen et al. [25] and showed that this technique eliminated the adverse effects of external fixation and reduced treatment time. However, a high rate of serious complications in patients treated by LATN was reported by Panagiotopoulou et al., [26] including deep infection, breakage of intramedullary nails and screws. As far as we are considering, the main drawbacks of the above techniques are the risk of internal fixator breakage and deep infection, limitation of DRL, instability, complexity of lengthening control device, and the huge burden of treatment costs. Therefore, there has been controversy regarding the choice of the above methods, which needs more evidence to support their clinical efficacy.

Based on the spectrum of multi-focal osteosyntheses of the Ilizarov technique, trifocal bone transport was firstly invented by Borzunov et al. [10] to smooth the way for the reconstruction of CSBD. The EFT can be shortened effectively by this technique while allowing the patient to mobilize on the 2nd postoperative day, accelerating bone mineralization dynamically [12–14, 27, 28]. Briefly, the duration of the distraction stage can be shortened half a time, and the DRL can be divided into equal parts [10, 11]. A case series of sixteen patients with tibial bone defects was published by Zhang et al. [29], and a satisfactory union was achieved after 6–7 months. Further, Li et al. [30] reported a total of 13 patients with tibial bone defects were successfully treated by trifocal bone transport using Orthofix external fixator, with a mean BUT of 8.93 ± 2.29 months. In this study, all patients were treated by trifocal bone transport using a unilateral external fixator, with a mean BUT of 334.6 days (range 317–347 days). Bone union was received in 39 patients (100%), and the rate of excellent and good bone and function was 74.3% and 87.1% respectively.

In our cohort, Staphylococcus (81.8%) were the predominant organisms responsible for the infection, followed by Enterobacteriaceae. We applied the first-generation cephalosporins was applied in the perioperative period since good Gram-positive coverage, combined with the intraoperative antibiotic-impregnated cement spacers for infection eradication. Besides, seven patients (17.9%) had different degrees of pin tract infection, including four cases in the femur group (22.2%) and three cases in the tibia group (14.2%). Similarly, the incidence of muscle contracture was also higher in the femur group than in the tibia. The complication of each patient was higher in the femur group than the tibia as well (femur vs tibia, 0.52 vs 0.44). Despite these complications being minor, more attention should be paid to these to prevent the failure of the whole treatment. In our experience, for the operative procedure, the principles of minimally invasive percutaneous osteotomy using Gigli saw should be followed to conserves the periosteum. The insertion of Schanz screws was recommended to conduct with the aid of a sleeve using a low-speed drill to reduce the incidence of necrosis by entangling the subcutaneous soft tissue.

Another phenomenon observed in this study was the high incidence of the delayed union in the tibia group (femur vs tibia, 5.5% vs 23.8%), which mostly occurred in patients with DRL > 8 cm, or previous surgery > 4 per patient. In our opinion, the vascular quality of the affected limb may be damaged by the previous surgeries and invasive debridement, which delayed the velocity of bone regeneration. Additionally, the anatomical structure changed in the middle and lower third of the tibial length [23], which resulted in fewer nutrient blood vessels. On the other hand, there was approximately related to a temporary imbalance between bone resorption and apposition when axial loading rapidly increased, which might cause few cortical layers to bridge the middle regenerate zone in the consolidation stage [31, 32]. Thus, the chief surgeon should fully understand the anatomical structure of the lower extremities and make a correct approach for the protection of the periosteum at the osteotomy site. Besides, the method was recommended by scholars that autologous cancellous bone grafting of the docking site at the end of distraction may effectively avoid the occurrence of delayed union [6, 33, 34]. In our cohort, the patients with the delayed union were managed by bone grafting and walking with the help of a walking aid after removal of the external clamps to achieve axial dynamization to enhance bone healing.

There were some limitations in this study. First of all, it was conducted retrospectively, and the chart review process may be subject to assessor bias. Secondly, results and complications showed in this study were a single center and a single surgeon case series at long-term follow-up. Thirdly, multi-centered trials with a larger sample size should be performed to assess the eventual efficacy of trifocal bone transport.

## Conclusion

In short, the trifocal bone transport, based on the Ilizarov technique, using the unilateral external fixator was a practical tool to manage the CSBD (> 6 cm) in lower extremities, whilst accompanying soft tissue defects simultaneously. In the comparison of the tibia, the BUT and EFI of the femur group were shorter. Although the complications noted were more frequent on the femur, these were mostly minor.

## Data Availability

The data sets generated and analyzed during the current study are not publicly available due to restrictions on ethical approvals involving patient data and anonymity but can be obtained from the appropriate authors as reasonably required.
